# Coreference Resolution Based on High-Dimensional Multi-Scale Information

**DOI:** 10.3390/e26060529

**Published:** 2024-06-19

**Authors:** Yu Wang, Zenghui Ding, Tao Wang, Shu Xu, Xianjun Yang, Yining Sun

**Affiliations:** 1 Hefei Institutes of Physical Science, Chinese Academy of Sciences, Hefei 230031, China; wy19910804@mail.ustc.edu.cn (Y.W.); wtustc@mail.ustc.edu.cn (T.W.); shuxu@mail.ustc.edu.cn (S.X.); xjyang@iim.ac.cn (X.Y.); ynsun@iim.ac.cn (Y.S.); 2 Science Island Branch, Graduate School of USTC (University of Science and Technology of China), Hefei 230026, China

**Keywords:** BERT, coreference resolution, high-dimensional features, multi-scale convolution, natural language processing, cross-entropy loss

## Abstract

Coreference resolution is a key task in Natural Language Processing. It is difficult to evaluate the similarity of long-span texts, which makes text-level encoding somewhat challenging. This paper first compares the impact of commonly used methods to improve the global information collection ability of the model on the BERT encoding performance. Based on this, a multi-scale context information module is designed to improve the applicability of the BERT encoding model under different text spans. In addition, improving linear separability through dimension expansion. Finally, cross-entropy loss is used as the loss function. After adding BERT and span BERT to the module designed in this article, F1 increased by 0.5% and 0.2%, respectively.

## 1. Introduction

Natural language processing (NLP) is a critical research area in computer science and Artificial Intelligence (AI). The language model is a fundamental component of NLP and plays a crucial role in predicting words or characters within a text sequence. Information theory has profoundly influenced this field, providing a mathematical framework dedicated to studying the quantification, transmission, encoding, and processing of information. This theory underpins the construction of language models and supplies essential concepts and methodologies for comprehending and manipulating textual data and encoding processes, among other linguistic operations.

**Figure 1:**
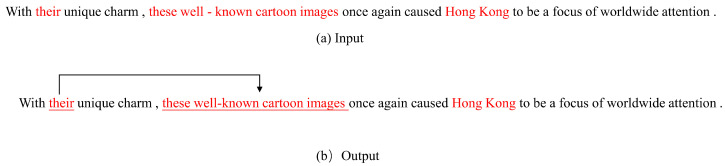
Example of a coref task. Red represents different entities and pronouns.

Pronouns can cause ambiguity in semantic understanding. Evidence suggests that language ambiguity is one of the most critical factors in how natural human language is represented by deep learning models [1]. Coreference resolution (coref) is the task of identifying mentions in a text that refer to the same entity or concept [2]. An example of a coref task is shown in Figure 1. Figure 1a illustrates the input for a coref task. When multiple entities are present in a sentence, the specific direction of the pronoun affects the machine’s understanding of the sentence. Figure 1b displays the output of the coref task, which assigns the pronouns in the sentence to the corresponding referential clusters and establishes associations with the correct entities. These associations can be utilized in downstream tasks to accurately understand the meanings of the corresponding pronouns.

This fundamental NLP task can benefit various applications, such as Information Extraction [3,4], Question Answering [5,6], Machine Translation [7,8], and Summarization [9,10], which are of great research value.

Coref requires document-level encoding. Evaluating the similarity of long-span texts presents a significant challenge in coref [11]. Neural network models are widely used in the field of computing. These models utilize word embeddings to capture word similarity, thereby effectively improving the accuracy of coref models. As a subtask of NLP, the coref algorithm based on deep learning faces the following challenges:

(1) Long-Span Problem: The issue at hand involves the span distance between pronouns and the entities to which they refer. In the traditional modeling process of deep learning language models, text lines of varying spans are often used as inputs. However, traditional sequence models, such as Recurrent Neural Networks (RNNs) and Long Short-Term Memory Networks (LSTM), often perform poorly and need improvement in capturing long-range dependencies. This deficiency occurs because their recursive calculation of long-distance information leads to information attenuation or loss;

(2) Ambiguous Reference: Ambiguous reference is a phenomenon in natural language processing where a pronoun or indicator may correspond to multiple potential entities within a single input. When multiple entities precede contemporary words, referential errors may occur. Resolving ambiguous references typically requires a detailed understanding of semantics and context, utilizing richer contextual, semantic and global information for recognition.

In recent years, various language models based on Transformers [12], such as BERT and GPT, have become the mainstream research focus to address the aforementioned issues. BERT employs a self-attention mechanism that effectively captures dependencies between distant words in a sentence. The multi-head attention mechanisms of the Transformer model, combined with BERT’s bidirectional encoding and context-sensitive embeddings, empower the model to capture dependency relationships and contextual information between words on a global scale. These methodologies significantly enhance the model’s capacity to understand contextual dependencies, thereby improving overall performance.

However, although BERT can capture rich contextual information within sentences, it still requires a more comprehensive global understanding for coref tasks. Therefore, challenges persist in using the BERT model to address issues related to long-span referencing.

The reasons for the above problems include the nature of the original feature maps in the BERT model, which exhibit low-dimensional features and excel at preserving many fine-grained details. However, a common challenge in coreference resolution tasks relates to long-span issues, where pronouns and their referents are far apart. It has been demonstrated that the limited range of low-dimensional feature maps in BERT does not provide adequate global information and spatial features, thereby hindering the model’s effectiveness in coref tasks.

Given the characteristics of the BERT model, several issues remain in improving and fine-tuning the model:

(1) BERT requires attention mechanism operations for each position within the original feature maps, which impedes the model’s ability to capture global information effectively under the constraints of large-scale feature maps;

(2) The original feature map is information-dense, and the size of the feature maps is large, which can obscure feature weights during model fine-tuning. This significantly affects performance and complicates the optimization process.

This phenomenon has led to traditional methods used in deep learning to acquire global information, such as sub-sampling and global average pooling, to potentially cause feature confusion. Consequently, this contributes to a decline in accuracy and presents challenges in further improving BERT’s capability to acquire global information.

Convolutional kernels of varying scales can capture different receptive fields, enabling the model to acquire more contextual and global information across multiple scales. This approach effectively addresses issues related to varying text spans and enriches the network’s contextual information [13]. However, Convolutional Neural Networks (CNNs) perform downsampling during the feature extraction stage to obtain more global features. This downsampling can obscure the features in BERT’s feature maps and negatively affect prediction results [14]. To leverage CNNs while overcoming the limitations of integrating them with BERT for coreference resolution tasks, this paper enhances the BERT model as follows:

(1) A new multi-scale convolution module is designed to process the feature maps and map the features to higher-dimensional spaces using convolution operations with different-scale convolution kernels. This module is then added to the BERT base model to obtain contextual information at different scales and to improve the sparsity of the features, thereby adapting to the problem of referring to different text spans;

(2) Given the substantial number of parameters in BERT, and because the convolution operation with a large-scale convolution kernel would significantly increase the number of parameters, this paper employs depth-separable convolution instead of regular convolution operations to reduce the computational load of the new module;

(3) The improved BERT model replaces the original BERT model among span-BERT and c2f-BERT configurations, and its performance is validated on the Ontonotes dataset [15], achieving better results than the original model.

## 2. Related Work

### 2.1. c2f-Coref

Lee et al. [16] devised the first state-of-the-art (SOTA) coref model, c2f-coref, which is an end-to-end learning system that utilizes only gold antecedent spans. This model combines a context-dependent boundary representation with a head-seeking attention mechanism and extracts text feature information using Bi-LSTM for extended embedding to perform reference disambiguation without relying on a grammar parser. Its results significantly outperform all previous work. Subsequently, Lee et al. [17] introduced a fully differentiable approximation to higher-order inference for c2f-coref to iteratively refine the span representation and softly consider multiple hops in the predicted clusters. The attention mechanism employs antecedent distribution from a span-ranking architecture to overcome the challenges of global feature selection. This model has been the foundation for reference resolution until now.

The core idea of c2f-coref is to treat the pronoun pairing problem as a probabilistic issue. It learns the conditional probability P by utilizing the product of polynomials configured to the most likely set that will yield the correct result. The probability is calculated as shown in Formula (1) below:

(1) $$P\left(y\right)=\frac{e^{s(x,y)}}{\sum_{y^{\prime }\in Y}e^{s(x,y^{\prime })}}$$

where s(x,y) is a span scoring function, expressed using a fixed length span.

With the span fixed, s(x,y) calculates the scoring function using a standard feedforward neural network, which is calculated as shown in Formulas (2)–(4) below:

(2) $$s\left(x,y\right)=s_{m}\left(x\right)+s_{m}\left(y\right)+s_{c}\left(x,y\right)$$

(3) $$s_{m}\left(x\right)=FFNN\left(gx\right)$$

(4) $$s_{c}\left(x,y\right)=FFNN\left(g_{x}\cdot g_{y},\emptyset \left(x,y\right)\right)$$

where $s_{m}\left(x\right)$, $s_{m}\left(y\right)$ are the output results of Bi-LSTM for both ends of the span, $s_{c}\left(x,y\right)$ is the joint compatibility score of *x* and *y*, FFNN(.) is the feedforward neural network, $g_{x}$ and $g_{y}$ represent its input, and ∅(x,y) denotes the speaker and metadata features.

### 2.2. Span-BERT

BERT has performed well across all areas of NLP, benefiting from its ability to model long-sequence feature vectors [18,19]. Researchers have applied it to coref tasks as well. Mandar Joshi et al. [20] replaced the LSTM module in the c2f-coref with BERT’s encoding module [21], designed the BERT-base coref model, achieving superior prediction results.

In further work, Mandar Joshi et al. [22] made the following modifications to the BERT-base coref model:

(1) Using the Mask Language Model (MLM), 15% of the tokens are randomly selected to be masked in the original training text, which allows the model to quickly learn the semantics of the token’s distributed context. This setup is not limited by the constraints of a one-way language model;

(2) Introducing the Next Sentence Prediction mechanism, which predicts the contextual relationships between sentences.

**Figure 2:**
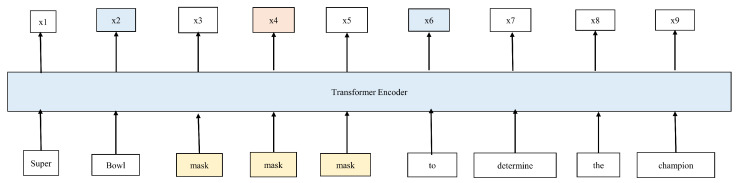
Span-BERT training for input span-BERT mask contiguous random spans as training input (in yellow), rather than random tokens (single word). SBO uses the output representations of the boundary tokens, *x*2 and *x*6 (in blue), to predict each token in the masked span. The middle layer uses a Transformer-based BERT encoding network for encoding. *x*4 (in orange) is the prediction result of the loss function using MLM and SBO. Since its development, its methodology has been widely used as a basic framework for subsequent improvements.

The introduction of BERT has dramatically improved model performance. Additionally, to enhance the model’s ability to infer citation relationships between two or more text spans, Joshi et al. developed the span-level pre-training model, span-BERT. Span-BERT masks units of spans rather than individual words. Subsequently, a Span Boundary Objective (SBO) module was introduced to characterize the content within the span as comprehensively as possible and to predict the tokens inside the masked span. This module encourages the model to learn the relational features between larger spans and improve the weighting of contextual relationship features within the mask. Thus, the model is better equipped to handle denotational disambiguation for long texts or multiple text spans. The structure of the span BERT model is illustrated in Figure 2.

The SBO-related network layers and loss calculations are depicted in Formula (5) as follows:

(5) $$\begin{array}{c} h0=f[x_{s-1};x_{e+1};P_{i-s+1}]\\ h1=LayerNorm(GeLU(\omega 1h0))\\ yi=LayerNorm(GeLU(\omega 2h1)) \end{array}$$

where $x_{s-1}$ represents the representation of the previous token at the beginning of the span, $x_{e+1}$ represents the representation of the next token at the beginning of the span, $P_{i-s+1}$ represents the $x_{i}$ position of the rowing pair in the mask, f(.) represents a two-layer feedforward network using Gelu activations and layer normalization, and yi is the output vector of SBO; this calculates the cross-entropy loss through yi, just like the MLM target. Weight matrices ω1 and ω2 are linear transformation matrices applied to the input vectors. They adjust the dimensions and introduce learnable parameters into the model.

The overall loss is shown in Formula (6):

(6) $$L\left(x_{i}\right)=L_{\mathrm{MLM}}\left(x_{i}\right)+L_{\mathrm{SBO}}\left(x_{i}\right)$$

where $L(x_{i})$ is the total loss and $L_{SBO}$$\left(x_{i}\right)$ and $L_{MLM}$$\left(x_{i}\right)$ represent the loss of SBO and MLM. Both use the cross-entropy loss function.

The coref task based on language models is often treated as a binary classification task. Entropy, a fundamental concept in information theory, is utilized to quantify the uncertainty within probability distributions. In the realm of language modeling, entropy serves as a metric for the uncertainty or randomness inherent in textual data. A language model with low entropy indicates a specific probability distribution of words or characters given a context, facilitating a more straightforward prediction of the subsequent word or character. Therefore, the cross-entropy loss function is widely applied in tasks such as coreference resolution.

Since coreference resolution is generally regarded as a binary classification problem, cross-entropy loss is used as the loss function for each part mentioned above. Leveraging BERT’s powerful performance, span-BERT (BERT-coref) has significantly improved on the public datasets Ontonotes and GAP [23], which are benchmarks for coref.

In span-BERT, researchers have also noted that the BERT model still shows limitations for long text and cross-document recognition [24,25]. Future research on pre-training methods should focus on using more sparse representations to encode document-level context more effectively. Based on this viewpoint, relevant personnel will continuously improve the BERT-based coref model in subsequent research to enhance performance.

Based on this perspective, relevant personnel will continuously improve the BERT-based coref model in subsequent research to enhance performance.

### 2.3. Other Related Work

To further reduce the computational cost of BERT models, Benjamin Hsu et al. [26] applied Contrastive Representation Learning. This technique involves training the model to distinguish between similar and dissimilar representations. By maximizing the agreement between different views of the same data point while minimizing the agreement between different data points, contrastive learning helps the model generate more efficient and robust embeddings. This approach reduces the need for extensive labeled data and improves the model’s ability to generalize from limited examples, thus lowering computational costs without sacrificing performance.

Additionally, Yuval Kirstain et al. [27] proposed a lightweight, end-to-end coreference resolution model (s2e + Longformer) that eliminates reliance on span representation and manual features. Their model leverages Transformer-based architectures to directly predict coreference links between text mentions. Instead of span-based features, the model uses contextual embeddings generated by the Transformer to represent mentions and their contexts. This approach simplifies the architecture and reduces the computational overhead associated with span-based methods, enhancing efficiency while maintaining high accuracy.

Shon Otmazgin and colleagues [28] developed a novel F-coref model based on the LINGMESS framework [29], which optimizes the architecture of multiple paired scorers to accommodate multilingual tasks and address diverse categories of coreferential instances. The F-coref model employs a set of specialized raters to assess referential pairs across various languages and syntactic structures. Each scorer is meticulously designed to handle specific linguistic phenomena and coreferential patterns, enabling the model to adapt to nuanced differences across languages. These scores are integrated into a cohesive framework that leverages local and global contexts to make coreference decisions. This modular approach enhances the model’s flexibility and accuracy in multilingual settings, improving performance across different language environments.

Kong et al. [30] created supervised fine-tuning (SFT) training data in camel format, as well as a set of low-rank adaptive (LoRA) weights, and developed a model that utilizes the principle of proportionality to obtain high-quality data. The model was fine-tuned to mitigate the influence of pronouns in group chat data.

Pražák et al. [11] developed an end-to-end model to address, for the first time, the coref problem without relying on manual features or independent-mention detection modules. The model introduces a mentioned head-prediction mechanism, which improves the effectiveness of coreferential resolution by identifying the keywords mentioned (i.e., the head) and inferring all span spaces up to the maximum length, thereby directly optimizing the marginal likelihood of the leading span from the gold coreference cluster. It includes a span-ranking model, determining which previous span is a good antecedent for each subsequent span.

Based on the BERT and span-BERT models, this paper makes further experimental improvements to the span prediction problem in coref, which has been applied to the coreference resolution problem of different text spans.

## 3. Model

The core module of the BERT model is the Transformer [31]. In 2017, Google introduced the Transformer model in the paper ‘Attention is All You Need’ [32]. The Transformer encoding module comprises a feedforward layer (FFNN) and multi-head attention. The multiple attention mechanisms consist of various groups of self-attention, with each self-attention mechanism responsible for establishing a separate feature matrix. The feedforward layer primarily integrates the feature matrices obtained from multi-head attention.

The self-attention mechanism is less dependent on external information and excels at capturing the internal relevance of data or features by computing the relationships between words, thereby overcoming long-range dependency issues. The relationships among the input tensors are extracted to obtain three tensors: *Q* (query), *K* (key), and *V* (value). Finally, the results are combined, and the self-attention is computed as shown in Formula (7) below:

(7) $$Attention\left(Q,K,V\right)=softmax\left(\frac{QK^{T}}{\sqrt{d_{k}}}\right)V$$

where *Q*, *K*, and *V* denote the weight matrices of the query, key, and value, respectively.

The value matrix *V* acts as an information carrier within the self-attention mechanism, encapsulating the actual content of the input sequence. During the attention calculation, the similarity between the query *Q* and the key *K* determines the weights applied to the value matrix *V*. This process identifies which parts of the information require more focus and extraction. The attention mechanism generates an aggregated output that represents the most pertinent information from the input sequence by performing a weighted summation of the value matrix.

Each self-attention computation produces the output of a distinct attention head. The outputs of all attention heads are concatenated and then subjected to a linear transformation to produce the final multi-head attention output, as illustrated in Formula (8) below:

(8) $$\begin{array}{c} MultiHead(Q,K,V)=Concat(head1,head2,\ldots ,headh)\omega 0 \end{array}$$

where head1,head2,…,headh represent multiple self-attention output results, ω0 represents the linear-layer weight matrix, and Concat represents the concatenation of results by dimension.

The self-attention mechanism reduces the model’s dependency on sequence length. The use of BERT provides an excellent solution to the problem of non-uniform natural language spans, and the span between the pronoun position and the subject to which it points cannot be precisely determined. Since BERT lacks a downsampling module, the original feature map is large-scale and dense, making global information less accessible. Conventional fine-tuning methods are also prone to feature weight confusion, leading to degradation in network performance.

CNNs are notably proficient in extracting local information but face challenges in acquiring global information. In the initial stages of research, the conventional method for acquiring global information involved a combination of subsampling and global average pooling. This method entails downsizing feature maps to capture global information. However, this approach is not suitable for enhancing the Transformer module, as the feature maps produced by the Transformer module are typically of a relatively larger scale. Downsizing these feature maps may lead to information loss and confusion.

In the efforts of other researchers, the pursuit of global information while preserving the integrity of local details is often addressed through the use of methods such as multi-scale convolutional sampling or spatial pyramid pooling. Multi-scale convolution leverages receptive fields of varying scales to effectively capture features across different spatial dimensions in the input data. This approach aids in the recognition and comprehension of both local and global information, thus enhancing the network’s robustness and generalization capabilities. It also helps mitigate overfitting issues in the network, improving its generalization capacity. Although this technique is less common in NLP, it is widely used in fields such as image segmentation.

**Figure 3:**
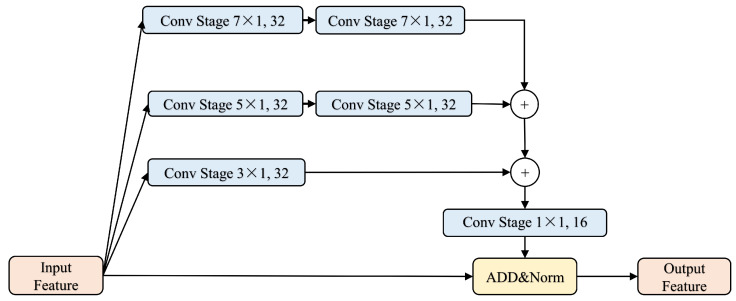
Multi-scale feature extraction module, where ConvStag is the depth-separable convolution operation of different scales: ConvStage7×1,32; ConvStage5×1,32; ConvStage5×1,32; and ConvStage1×1,16, where 7×1 and the like represent the size of the convolution kernel, and 32 and 16 represent the number of channels, i.e., the feature dimension. + is a concat, ADD is an addition operation, and Norm is batch normalization.

To enhance the performance of BERT, this paper proposes a novel multi-scale convolutional model based on the downsampling module in Feature Pyramid Attention (FPA) [33,34]. This model convolves the input vector with kernels of various scales to capture richer contextual information. Given the downsampling operations in CNNs, which can obscure the feature weights of the extracted feature maps, this paper adopts a parallel operation approach from Inception [35,36] to simultaneously extract multi-scale feature information, thereby counteracting the adverse effects of downsampling. Additionally, considering the numerous hidden layers in BERT, parallel operations help prevent the gradient vanishing problem associated with excessive network depth. The architecture of the multi-scale feature extraction module is depicted in Figure 3.

Firstly, the feature dimension is increased through a convolution operation on the input features. After processing with convolution kernels of different scales, the dimension is reduced back to that of the input feature through dimension-independent splicing. This is followed by adding the input feature to the output. The convolution operation with kernels of various scales allows the capture of different scales of receptive fields, which provide diverse scales of feature information for the model and enhance the capture of global contextual associations. The dimension-raising operation, conducted during convolution, maps the feature map to a high-dimensional space, improving the sparsity and, to some extent, alleviating the issue of overly dense feature maps extracted by the BERT model. This is followed by a dimensional splicing operation, then a dimensional reduction to restore the original dimensions and a channel shuffle to select the appropriate scale features while reducing redundancy. Finally, the obtained multi-scale features are combined with the original features for output.

**Figure 4:**
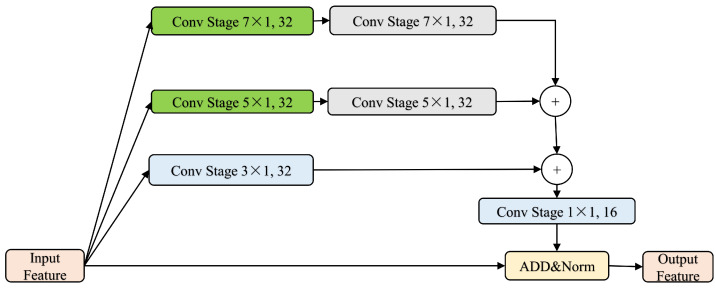
Multi-scale, where + is a concat, ADD is an addition operation, and Norm is batch normalization. feature extraction module with down-sampling. Green represents a downsampling operation during the convolution process, gray represents upsampling, and blue represents unchanged feature map size.

**Figure 5:**
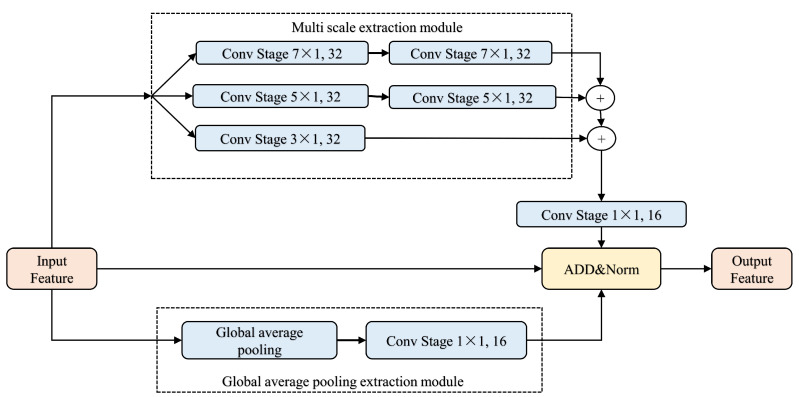
Multi-scale feature extraction module with global-average pooling, where ConvStage is the depth-separable convolution operation of different scales, + is a concussion, ADD is addition operation, Norm is batch normalization.

Other commonly used design schemes to enhance the network’s capability to obtain global information are illustrated in Figure 4 and Figure 5. Figure 4 depicts a downsampling operation scheme, and Figure 5 illustrates a scheme incorporating global-average pooling information. These two design schemes are used for comparative experiments to assess the impact of improving global-average pooling on Transformer performance.

Given BERT’s already large number of parameters, and considering that convolution operations with large convolution kernels can significantly increase the model parameters, this paper employs depth-separable convolution and light-weighting techniques to mitigate the increase in parameter count.

**Figure 6:**
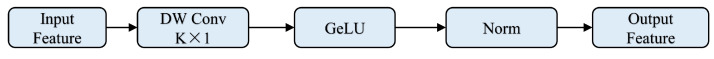
Conv Stage, where Norm is batch normalization, Gelu is the activation function used in BERT, DWConv *K* ×1 is the depth-separable convolution of convolution kernel *K* ×1. After the depth-separable convolution, the input feature is activated by Gelu once and finally output by a linear transformation.

The Conv Stage flow is shown in Figure 6 below.

This paper applies the aforementioned multi-scale feature extraction module to the multi-head attention process. Given the distinct contents processed in the Q, K, and V feature vectors within the self-attention mechanism, only V is processed in this manner, as detailed in Formula (9) below:

(9) $$\begin{array}{c} F\left(V\right)=[f^{7\times 1}\left(V\right);f^{5\times 1}\left(V\right);f^{3\times 1}\left(V\right)]\\ h1=LayerNorm(GeLU(F(V)))\\ O(v)=h1+V \end{array}$$

where $f^{k\times 1}$ is the convolution operation, LayerNorm is batch normalization, and Gelu is the activation function used in BERT. Since *V* is in the form of a four-dimensional tensor, the convolution operation of *K* ×1 is used here for only one dimension. *h* ×1 represents the activation layer and normalization layer that have been passed through once.

**Figure 7:**
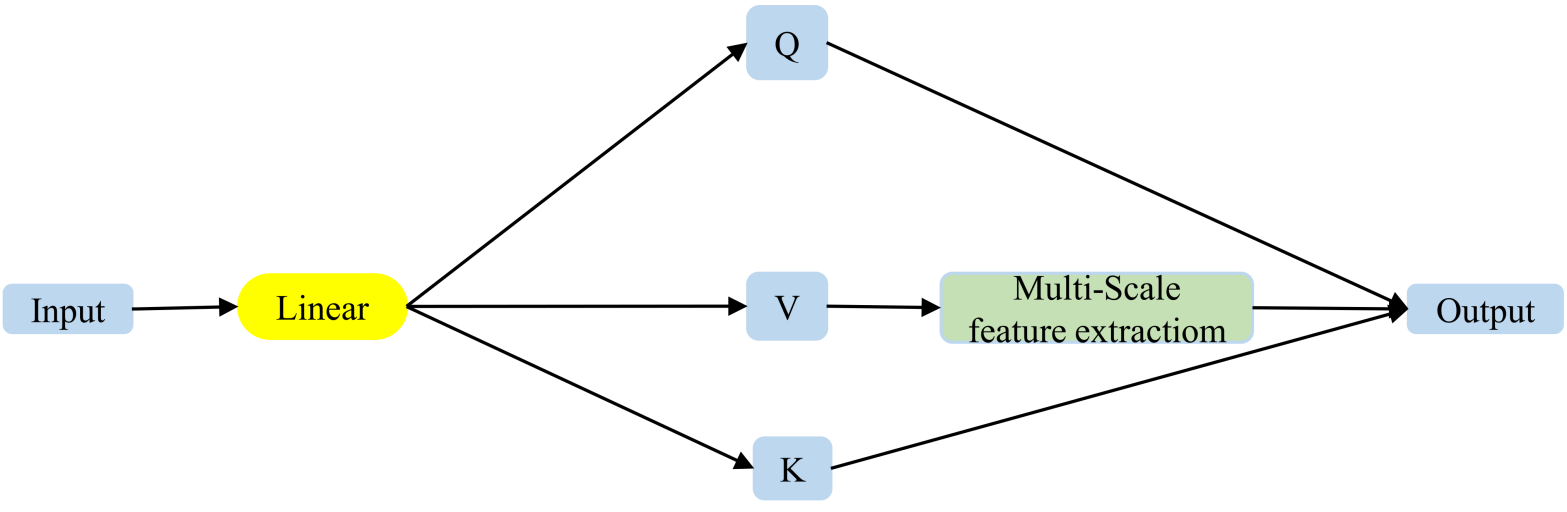
Improved multi-head attention. This paper uses the multi-scale feature extraction module to improve the computation of multi-head attention mechanism. After linear transformation, the input features obtain three feature tensors *Q*, *K*, and *V*. For *V*, we use the multi-scale feature extraction module for further processing and then calculate attention.

The specific usage is shown in Figure 7 below.

Basic BERT employs an improved multi-head attention mechanism. Cross-entropy loss was used as the primary loss function in constructing the final loss function. The multi-scale feature extraction module was validated on the Ontonotes dataset and achieved notable enhancement effects.

## 4. Experiment

### 4.1. Datasets and Parameters

This paper utilizes the document-level dataset Ontonotes (English) from the CoNLL-2012 [37] shared task of coreference resolution. The dataset comprises 2802 documents, including 343 training documents and 348 test documents, and contains approximately 1 million words spanning newswire, broadcast news, broadcast conversations, and web data. The primary evaluation employed the official CoNLL-2012 evaluation script to test three metrics from the test set: average F1-MUC, B3, and CEAFφ4.

**Figure 8:**
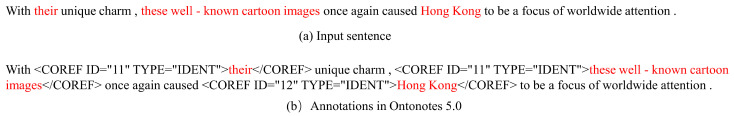
Ontonotes original annotated document. Red represents the entities and referents that need to be identified.

Figure 8 below presents the original annotation document for coref tasks in Ontonotes 5.0.

Figure 8a displays an example of the input text, and Figure 8b shows an annotated document. During the annotation process, it is essential to identify the beginning and end positions of entities or pronouns and annotate their refers cluster IDs.

**Figure 9:**
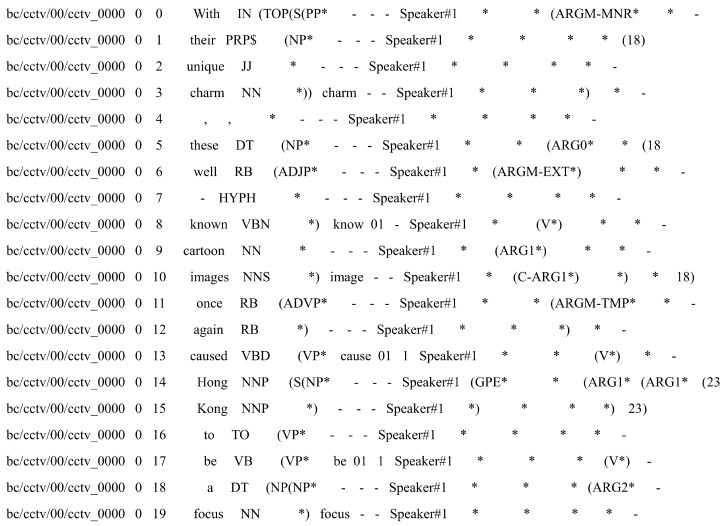
Conll-2012 data format annotation.

Before performing the coref task on the original Ontonotes 5.0 data, it is necessary to preprocess the data using the relevant code provided by Conll-2012. The preprocessing results are shown in Figure 9, where Figure 9 demonstrates the conversion of the Ontonotes 5.0 original annotation to the Conll-2012 standard annotation format. Each column in the annotation format represents the file name, sentence number, word index, part of speech, sentence structure information, speaker, referential cluster, and other sequential information.

**Figure 10:**
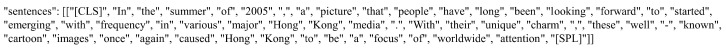
Input format [CLS] represents the beginning of a sentence; [SPL] represents a placeholder for punctuation.

When using the above annotation file as input for the coref model, it is also necessary to convert it to the JSONLINE format shown in Figure 10. Each word is treated as a token, and the annotation file is reorganized into the annotation file based on the set maximum span (ensuring the integrity of the sentence). Each span is then used as input.

The input document is encoded and trained by the model to obtain the model. In the test set, based on the trained model and annotated reference cluster labels, it determines whether each pronoun is assigned to the correct reference cluster and finally outputs the total evaluation parameter results for all test files.

The server has two 12th Gen Intel(R) Core (TM) i9-12900K processors, each with a clock frequency of 3.20 GHz, was manufactured by Intel Corporation in Oregon, USA, and a total memory capacity of 64 GB RAM. Additionally, the server is configured with two NVIDIA GeForce RTX 3090 GPUs, each with 24 GB of RAM. The NVIDIA GeForce RTX 3090 GPUs are manufactured by NVIDIA Corporation. The company’s headquarters are located in Santa Clara, CA, USA.

The experiment was configured with a dropout rate of 0.3, learning rates of $1\;\times \;10^{-5}$ and $2\times 10^{-4}$, and a linear decay rate of 0.1. A maximum of the first 50 antecedents were selected for analysis (max_top_antecedents = 50). The maximum number of sentences used during each training session was 5, with the first 40% of the span selected for analysis (top_span_ratio = 0.4), and a maximum of 20 different speakers were considered (max_num_speakers). The hidden layer size of the feedforrward neural network was set to 1000, with two hidden layers (ffnn_size = 1000, ffnn_depth = 2). The training was conducted over 30 epochs (num_epochs = 30), with a feature dimension of 20 (feature_size = 20) and a maximum reference span of 30 (max_span_width = 30). The Adam optimizer was used, with a decay rate of adam_eps at $1\times 10^{-6}$. Additionally, testing experiments on the BERT base and span model were conducted under the maximum segmentation lengths of 128 and 256 segments, respectively.

### 4.2. Results and Analysis

This paper initially evaluates the impact of global-average pooling, downsampling, and multi-scale convolution on the coref task based on the BERT model. These techniques are commonly employed to enhance semantic information. However, as indicated by the results presented in Table 1, applying global-average pooling and downsampling to BERT models for coref tasks can introduce perturbations in feature vectors, resulting in a degradation of network performance. Therefore, these techniques are deemed unsuitable for referent resolution tasks.

Specifically, the F1 score of the BERT-base model is 73.9%, while the models utilizing global-average pooling and downsampling have F1 scores of 55.8% and 64.6%, respectively, which are significantly lower than the base model. This indicates that the global-average pooling and downsampling methods may underperform in this task, possibly due to the loss of critical semantic and contextual information during feature extraction.

Conversely, employing multi-scale convolution to amalgamate local information from different receptive fields enables the network to acquire additional contextual information, thereby facilitating a more comprehensive understanding of the input data. The model with multi-scale convolution achieves an F1 score of 74.4%, slightly higher than the base BERT model. This suggests that, by integrating information from multiple scales, the model can capture semantic and structural features at various levels, thus enhancing its ability to understand the input data.

In further ablation experiments, this paper investigated whether dimension enhancement affects network performance, specifically the impact of changes in the dimension of intermediate feature maps on model performance during convolution operations. As depicted in Table 2, the results indicate that, without dimensionality augmentation, the simple convolutional operations on the dense feature maps of the BERT model necessitate weighted computation of adjacent feature maps. This process results in a decline in the linear separability of the model, leading to a reduction in the F1 score to 73.9%.

On the other hand, dimensionality expansion enhances the linear separability of feature maps while preserving more original feature information, resulting in an improved F1 score of 74.4%. This improvement can be attributed to dimensionality expansion, which allows the model to handle the feature maps more effectively by capturing and preserving essential features.

These findings suggest that dimensionality augmentation enhances the performance of BERT models. By improving the linear separability of the feature maps and preserving more original feature information, dimensionality expansion allows CNNs within the BERT model framework to process the dense feature maps more efficiently, thereby alleviating the challenges associated with straightforward convolutional operations on unaltered dimensionality.

The results in Table 3 indicate that the module developed in this paper increases the average F1 score by approximately 0.5% for the original BERT-based models and by 0.2% for the span-BERT models. This improvement signifies a positive impact of the module on BERT models, particularly in enhancing precision.

The multi-scale convolutional kernel’s operation enables the model to access contextual information at various scales, which is beneficial for feature screening. This enhanced feature screening improves the model’s precision, albeit with a slight decrease in recall, which accounts for the modest overall improvement in F1 score.

For example, the BERT-based + ours (IND) model slightly raises the average F1 score from 73.9% to 74.2%, while the BERT-based + ours (Ovlp) model shows an increase from 73.9% to 74.4%. Similarly, the span-BERT + ours model records an improvement from 79.6% to 79.8%. These results confirm that the designed module effectively manages the feature maps and captures essential information, thus improving model performance, especially in terms of precision.

This paper also conducted experiments using BERT (independent, IND), BERT-based (overlapping, OVLP), and span-BERT as baselines, testing the efficacy of the improved model under different input-text segmentation lengths. Two commonly used segmentation lengths, 128 and 256, were selected for testing. As detailed in Table 4, the proposed model demonstrates an improvement in F1 score by 0.2% at a segmentation length of 128 and by 0.5% at a segmentation length of 256. This suggests that the model performs better with longer segmentation lengths, providing a broader context for feature extraction, which leads to improved overall model performance.

The observed improvement may be attributed to the fact that, with longer segmentation lengths, the span of the subject to which the word points becomes more uncertain. In such cases, the multi-scale receptive field can more effectively capture the necessary features to make accurate judgments. The ability of the multi-scale convolutional kernel to acquire contextual information at various scales proves beneficial in these scenarios, as it enhances the model’s capability to handle longer and more complex sequences.

For example, at a segmentation length of 256, the proposed model’s F1 score increases from 73.9% to 74.4%, indicating a significant performance enhancement. This suggests that the multi-scale approach is particularly effective in scenarios where the segmentation length is substantial, likely because it provides a broader context for feature extraction, leading to better overall model performance.

As can be seen from Table 5, the module proposed in this paper can effectively improve the efficiency of the BERT-based model and has achieved better results in the related BERT-based model improvement results.

## 5. Conclusions

Leveraging its robust encoding capabilities, BERT has achieved groundbreaking successes across various domains of NLP. Models such as BERT-coref have effectively applied BERT in semantic disambiguation, demonstrating notable efficacy. However, challenges persist in BERT’s application to coreference resolution, particularly due to discrepancies in pronoun lengths and subject spans within discourse. Enhancing the contextual and global information acquisition capabilities of encoding modules holds promise for addressing these issues, suggesting a direction for future enhancements to further improve BERT’s performance in complex NLP tasks.

Based on the BERT model, this paper evaluates the impact of downsampling, global-average pooling, and multi-scale convolution on the coreference resolution task. Multi-scale convolution, in particular, proves especially advantageous in enhancing network performance. Building upon this observation, we have devised a convolutional operation module equipped with multi-scale receptive fields. This module enables the model to extract contextual information independently of text length, facilitating refined text-span selection. Additionally, projecting features into higher-dimensional spaces during processing aids in acquiring sparser feature representations, which are beneficial for the model’s performance. We also employ cross-entropy loss as the training objective. Experimental findings demonstrate the augmented model’s superiority over its precursor, although improvements are less conspicuous when text truncation lengths are excessively short.

## Figures and Tables

**Table 1:** Effects of diverse approaches for enhancing global information retrieval capabilities on the BERT network for coref tasks.

Model	F1
BERT-based	73.9
Global-average pooling	55.8
Downsampling	64.6
Multi-scale convolution	74.4

**Table 2:** Comparison of the results of the BERT-based model with different-dimension operations.

	F1 (BERT-Based + Ours)
No change in dimensionality	73.9
Dimensionality expansion	74.4

**Table 3:** Comparison of the experimental results of the modules designed in this paper added to the BERT model.

	MUC			B3			CEAF*φ*4			
	P	R	F1	P	R	F1	P	R	F1	Avg. F1
BERT-based (IND)	80.2	82.4	81.3	69.6	73.8	71.6	69.0	68.6	68.8	73.9
BERT-based + ours (IND)	81.0	81.8	81.4	71.0	73.2	72.1	71.5	66.7	69.1	74.2
BERT-based (Ovlp)	80.4	82.3	81.4	69.6	73.8	71.7	69.0	68.6	68.8	73.9
BERT-based + ours (Ovlp)	80.5	82.7	81.6	72.5	71.0	73.0	71.0	66.2	68.6	74.4
Span-BERT	85.8	84.8	85.3	78.3	77.9	78.1	76.4	74.2	75.3	79.6
Span-BERT + ours	86.0	84.8	85.4	78.5	78.3	78.4	78.2	74.0	76.1	79.8

**Table 4:** Comparison of the results of the BERT-based model with different segmentation lengths.

Seg-Len	F1 (BERT-Based)	F1 (BERT-Based + Ours)
128	74.4	74.6
256	73.9	74.4

**Table 5:** Comparison of the results of the BERT-based model with recent related work.

	MUC			B3			CEAF*φ*4			
	P	R	F1	P	R	F1	P	R	F1	Avg. F1
span-BERT + ours	86.0	84.8	85.4	78.5	78.3	78.4	78.2	74.0	76.1	79.8
F-coref	78.5	84.3	81.3	68.2	74.8	71.4	64.1	72.9	68.2	73.7
c2f + Longformer	80.6	83.2	84.6	78.9	75.5	77.2	76.7	68.7	72.5	78.1
s2e + Longformer	86.5	85.1	85.8	80.3	77.9	79.1	76.8	75.4	76.1	80.3

## Data Availability

This article uses the Ontonotes 5.0 dataset for experimentation (https://www.ldc.upenn.edu/) and the right to use the data has been obtained on 14 December 2022.
